# Potential carbon storage in biochar made from logging residue: Basic principles and Southern Oregon case studies

**DOI:** 10.1371/journal.pone.0203475

**Published:** 2018-09-13

**Authors:** John L. Campbell, John Sessions, David Smith, Kristin Trippe

**Affiliations:** 1 Department of Forest Ecosystems and Society, Oregon State University, Corvallis, Oregon, United States of America; 2 Department of Forest Engineering, Resources and Management Oregon State University, Corvallis, Oregon, United States of America; 3 Department of Wood Science and Engineering, Oregon State University, Corvallis, Oregon, United States of America; 4 USDA Agricultural Research Service NSFPRC, Corvallis, Oregon, United States of America; RMIT University, AUSTRALIA

## Abstract

The industrial production of long-lived charcoal products (commonly referred to as biochar) from otherwise shorter-lived logging resides (commonly referred to a slash) has been proposed as a means to increasing terrestrial carbon storage thus mitigating global warming caused by anthropogenic greenhouse gas emissions. We present a generalized model that describes the temporal dynamics of biochar carbon stocks, relative to carbon of unmodified logging residue, and evaluate the sensitivity of carbon storage to various biophysical and production parameters. Using this model, we then attribute net carbon storage to several potential biochar production scenarios, specifically engineered to use wood recovered from harvests prescribed to reduce fire hazard in mixed-conifer forests of South-central Oregon. Relative to a baseline scenario where logging residue is left to decay on site, the net carbon storage attributed to 20 years of biochar production is generally negative for the first several decades, then remains positive for several centuries at levels approximately one-fourth the total feedstock carbon processed. Positive net carbon storage and the time required for it to manifest is notably sensitive to biochar conversion efficiencies, logging residue decay rates, and alternate baseline fates of logging residue. The magnitude of net carbon storage, and the time required for it to become positive, is largely similar across range of production facility types. Moreover, the time required for net carbon storage to become positive, and its magnitude over the first 100 years is notably insensitive to biochar decomposition rates provided biochar decays at least ten-times slower than the logging residue it is made from.

## Introduction

The pyrogenic conversion of short-lived agricultural residues into various long-lived charcoal products, commonly referred to a biochar, has been proposed as an effective means to increase terrestrial carbon storage, thus mitigating global warming caused by anthropogenic greenhouse gas emissions [1,2,3]. Concurrent to terrestrial carbon storage, biochar produced from agricultural residues has the potential to yield renewable energy [4,5,6] and, as a soil amendment, increase water-holding capacity and crop production [7,8]. Realizing the ecological benefits of biochar depends on many factors ranging from the carbon dynamics that describe storage potential, the physical and microbial processes that define soil fertility, the regulatory policies that incentivize climate mitigation, and the market forces that dictate the profitability of biochar production. Consequently, quantifying the benefits and feasibility of large-scale biochar production requires a clear understanding of both the biophysical principles constraining potential outcomes and comprehensive case studies describing the likely outcome of real-world biocharring operations from feedstock production, through industrial pyrolysis, to the end-uses of biochar. The purpose of this paper is to address the feasibility and potential carbon consequences of converting logging residue into biochar and incorporating that biochar into soils supporting food crops in a semi-arid region of western North America.

The removal of small-diameter trees in forests considered artificially dense due to decades of fire suppression can be effective in reducing vertical fuel continuity and thus the likelihood that remaining trees will die when exposed to certain wildfire behavior [9]. Commonly referred to as fuel-reduction treatments, the logging of relatively young, small-diameter trees in fire-prone forests is advocated by many as necessary to restore fire-resilient structure to native forests believed to have evolved in high-frequency, low-severity fire regimes [10,11]. The portion of logged trees left behind on site after the commercial components are removed are termed logging residues (also sometimes called slash). Logging residues are comprised of branches, tree tops, defective parts, breakage, and noncommercial species. In areas where commercial pulpwood markets do not exist, logging residues may also contain logs with large end diameters smaller than 15 cm. Because the trees typically targeted for removal in fuel-reduction treatments are small-diameter, any of the fuel-reduction treatments thought necessary to restore ecosystem resilience to fire-prone forests, are hampered by operational costs which exceed the value of harvested wood. One popular solution to this dilemma is to use the logging residue generated in fuel-reduction treatments as feedstock for power generation plants [12,13,14] or convert it into liquid biofuels [15,16]. These solutions are attractive in part because the energy market is theoretically large enough to support the large-scale forest restoration efforts advocated by some forest managers, and in part because the use of logging residue to offset fossil fuel combustion can, over long time frames, compensate for the terrestrial carbon losses associated with logging [17,18]. The production of biochar from logging residue generated in fuel-reduction treatments represents a new opportunity to use wood as a feedstock, perhaps leading to carbon and economic benefits exceeding that of energy production alone. Because biochar itself represents a carbon pool with greater longevity than its source wood, the carbon sequestration benefits of biochar are more tangible than that of fossil offsets. Furthermore, the potential for wood-derived biochar to improve the health and fertility of agricultural soils, while continuing to serve a long-term carbon reservoir, may confer biochar higher market and environmental value than the energy produced from either the combustion or digestion of wood waste.

To evaluate the feasibility and potential carbon consequences of converting low-value wood into biochar, this paper is divided into four sections. First, we present a generalized model that describes the temporal dynamic of biochar carbon stocks relative to carbon of unmodified logging residue. Using this model, we evaluate the sensitivity of carbon storage to basic biophysical parameters including decay rates and biochar conversion efficiencies. Second, we consider the sensitivity of carbon storage to various biochar production parameters including: production rate, project duration, carbon emissions associated with feedstock recovery and handling, carbon offsets attributed to energy capture, and the potential for biochar, once added to soil, to immobilize and or accelerate decomposition of native soil organic matter. Third, we evaluate the net carbon storage which can be attributed to biochar relative to various alternate baseline scenarios including those where logging residue is left unmodified on site, chipped and left on site, deliberately burned on site, or left on site and subject to wildfire. Finally, we conduct a comprehensive carbon accounting of several potential forest-to-farm biochar operations–employing either conventional pyrolysis or microwave technology–specifically engineered to use wood recovered from fuel-reduction harvests prescribed for mixed-conifer forest of South-central Oregon and subsequent incorporation of that biochar into nearby agricultural soils. Together, we use these generalized and project-specific models to describe the potential long-term carbon consequences of producing biochar from the thinning of fire-prone forests, and identify the parameters most critical for achieving carbon storage goals.

## Methods

### Describing basic system behavior and sensitivity to biophysical parameters

The carbon storage attributed to biocharring was calculated as the difference between the mass of carbon residing in the form of biochar, and a baseline mass of carbon that would have otherwise resided in the form of logging residue, had it not been converted into biochar, according to Eq 1:

$$NCS=C_{biochar}-C_{residue}$$ (1)

where, *NCS* is the net carbon storage attributed to biocharring in any given year (y-axis for all figures in this paper); *C*_*biochar*_ is the mass of manufactured biochar carbon persisting in that year; and *C*_*residue*_ is the alternate baseline mass of unmodified logging residue carbon persisting in that same year. The mass of carbon present in both the biochar and alternate unmodified residue pools were modeled dynamically at annual time steps according to Eq 2:

$$C_{t}=C_{t-1}\;e^{-k}+C_{input}$$ (2)

where *C*_*t*_ is the mass of carbon present in either biochar or unmodified logging residue in any given year; *C*_*t-1*_ is the mass of carbon present in that pool one year earlier; *k* is a first order exponential decay constant specific to either biochar or unmodified logging residue (expressed in yr^-1^) describing carbon loss lost through metabolic decomposition, from terrestrial storage, to the atmosphere; and *C*_*input*_ is the mass of carbon added to the pool each year. For the baseline pool of logging residue, *C*_*input*_ is simply the amount of logging residue carbon generated annually. For the biochar pool, *C*_*input*_ is an equal amount of carbon, reduced by the fraction combusted during pyrolysis (i.e. the biochar conversion efficiency).

**Fig 1:**
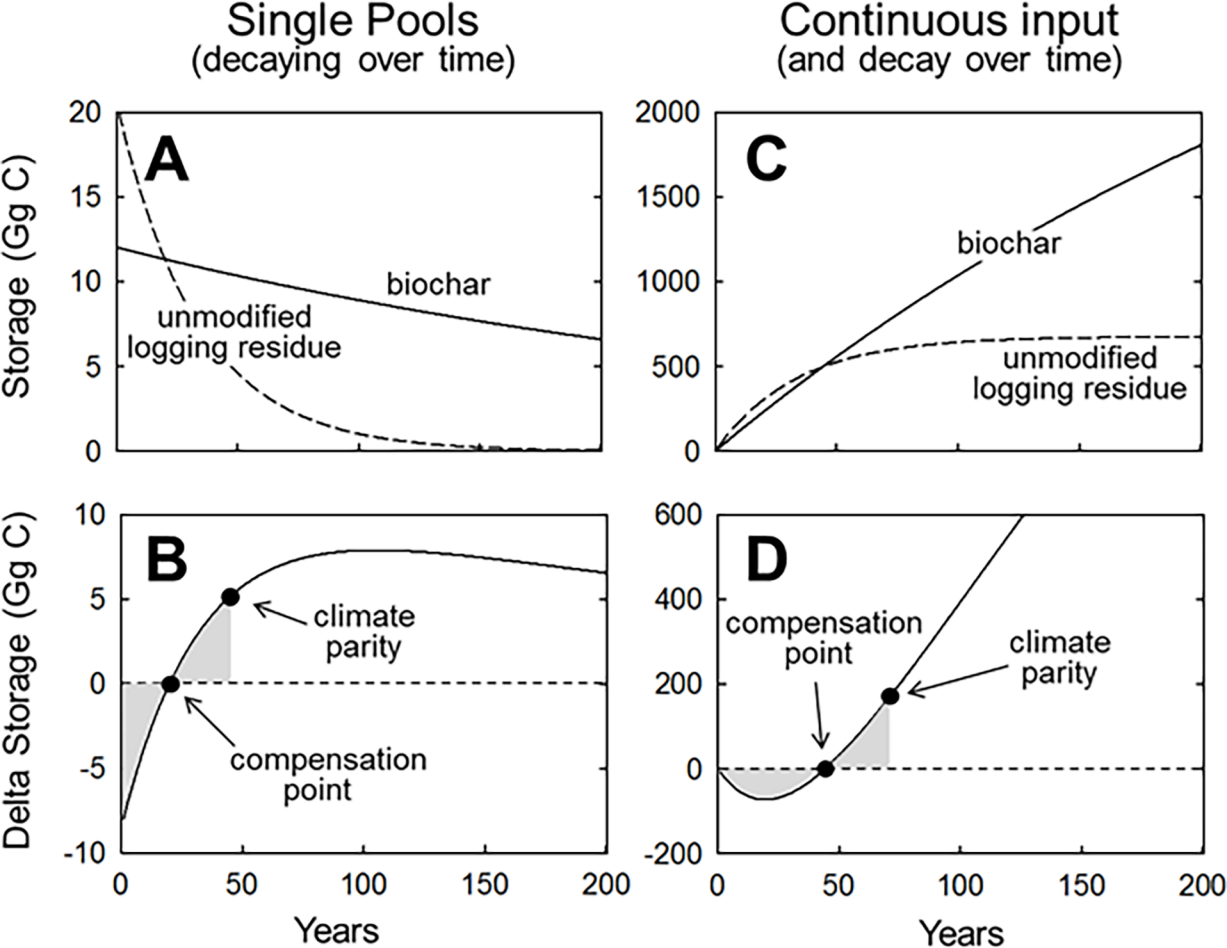
Basic system behavior illustrating the carbon storage benefits of converting logging residue into biochar. In these examples, biochar is assumed to lose 40% of its carbon to the atmosphere during production and the decay rate is assumed to be one-tenth that of unmodified logging residue. **A** and **B** show the consequences of converting a single pool of unmodified logging residue into biochar. **C** and **D** show the consequences of converting a perpetual supply of unmodified logging residue into biochar. The *compensation point* is when the carbon stored in biochar is equal to that which would have been stored in logging residue if left unmodified (when Δstorage = zero). *Climate parity* is when the amortized carbon storage attributed to biochar equals the amortized carbon debt incurred prior to the compensation point (when shaded areas above and below the Δ = zero line are equal). Only after climate parity can it be said that climate change mitigation is occurring. For simplicity, potential carbon costs or gains attributed to energy consumption or generation during biochar production are not included in these examples.

In this study, we define the compensation point as the year in which *NCS* first becomes positive and climate parity as year in which the cumulative positive *NCS* (incurred following the compensation point) first equals the cumulative negative *NCS* (incurred prior to the compensation point). For a single pool of biochar, the compensation point is reached when the conditions of Eq 3 are first met, and climate parity is reached when the conditions of Eq 4 are first met:

$$C_{0}\left(e^{‑k1t}\right)=f\;C_{0}\left(e^{‑k2t}\right)$$ (3)

$$\int_{0}^{T_{1}}C_{0}e^{-k_{1}t}-fC_{0}e^{-k_{2}t}dt=\int_{T_{1}}^{T_{2}}fC_{0}e^{-k_{2}t}-C_{0}e^{-k_{1}t}dt$$ (4)

where, *C*_*0*_ is the initial carbon made available as residue; *k*_*1*_ and *k*_*2*_ are the first order decay rates of unmodified residue and residue-derived biochar, respectively; *f* is the carbon conversion efficiency (i.e. the fraction of original carbon left after pyrolysis); *t* is time elapsed since the simultaneous creation of both the residue and biochar pools; and *T*_*1*_ is the compensation point and *T*_*2*_ is the point of climate parity (see later in Fig 1B).

While simulating decay from single pools of biochar and residue is helpful in understanding the underlying dynamics, a more applicable model considers a continuous generation and decay of biochar and residue (see later in Fig 1D). In this case of continuous input, the compensation point *T*_*1*_ is reached when the conditions of Eq 5 are first met:

$$C_{\mathrm{input}}\;\sum_{t=0}^{T_{1}}e^{-k_{1}t}=fC_{\mathrm{input}}\;\sum_{t=0}^{T_{1}}e^{-k_{2}t}$$ (5)

where *C*_*input*_ is the production of residue carbon per unit time. In this case of continuous input, climate parity is reached when the conditions of Eq 6 are first met (see also Fig 1B):

$$\sum_{i=0}^{n}I_{i,T_{1}+i}=-(\sum_{i=0}^{n}I_{T_{1}+i,T_{2}+i}-\sum_{j=0}^{n-1}I_{T_{1},T_{1+n},j})$$ (6)

where *T*_*1*_ is equal to the single-pool compensation point (according to Eq 3) and *I*_*input*_ is defined according to Eq 7.

$$I=\int_{0}^{T_{1}}C_{0}e^{-k_{1}t}-fC_{0}e^{-k_{2}t}dt$$ (7)

For the purpose of these exercises, the carbon concentration of unmodified dry logging residue was assumed to be 0.5 (reliable approximation for all woody tissue; see [19]) while the carbon concentration of biochar produced from the same residue was assumed to be 0.75 (as reported for conifer-derived biochars; [20,21,22].

To evaluate the consequences of decay rates on net carbon storage over time, we considered logging residue decay rate constants of 0.01, 0.03, 0.06, and 0.09 yr^-1^, (half-lives of 70, 23, 12, and 8 years, respectively) each paired with biochar decay rate constants of either 0.5, 0.1, 0.01, 0.001 times the logging residue decay rate. These logging residue decay rates served to bracket various empirical estimates of fine woody debris decay in fire-prone forests which average approximately 0.03 [23,24,25]. Similarly, the range of biochar decay rates served to bracket a variety of empirical and theoretical estimates of biochar recalcitrance, wherein biochars commonly are described as having decay rates 10 to 1000 times slower than their parent feedstock, depending in part on chemical composition and in part on decay environment [26]. To evaluate the consequences of biochar conversion efficiencies (i.e. biochar C per unit feedstock C) on net carbon storage over time, we considered carbon mass yields of 0.4, 0.5, 0.6, and 0.7, which, given the above-mentioned carbon concentrations of logging residue and biochar, translate to a dry mass yield of 0.27, 0.33, 0.40, and 0.47, respectively. It is worth noting that when producing biochar from wood, 10–20% of the volatized carbon may re-condense in the form of acetic acid (wood vinegar) and or creosotes (wood tar). The fate and longevity of these carbon pools varies hugely depending on the production facilities and market opportunities. For the purposes of this analysis, such biochar by-products are considered as losses from the system.

### Sensitivity to biochar production parameters

Using the model described above, we considered the effects of production rate, project duration, and various additional carbon costs and benefits of a forest-to-field biochar operation. Specifically, we assumed a 4% carbon cost attributed to feedstock transportation and processing, a 2% carbon gain through energy capture and fossil fuel offset, and a 10% carbon gain attributed to negative soil priming (i.e. the immobilization of native soil carbon resulting from the deliberate incorporation of biochar). The carbon costs and gains attributed to energy consumption and production were approximated from operational case studies described below, while the carbon gains attributed to soil priming is meant to approximate the upper end of biochar immobilization capacity reported by others [27].

### Alternate baselines

The carbon consequences of any intervention can be quantified only with respect to some alternate baseline scenario. In this study, the default baseline to which we compare biocharring is a no-action leave-and-decay scenario, wherein logging residue generated from forest thinning is left on site to decay, unmodified. However, many forest restoration operations designed to reduce fire hazard do involve additional treatment of residue designed to reduce the spread rate and flame length of subsequent fires, all of which reduce *in situ* carbon storage. To evaluate the consequences of biocharring relative to these alternate baseline actions, we simulated the difference in carbon storage over time between a common, mid-range biocharring scenario and the following alternate baselines: 1) *a pile-and-decay scenario*, as used in the earlier sensitivity analysis; 2) *a scatter-and-decay scenario*, wherein logging residue decays somewhat faster due to greater ground contact; 3) *a mulch-and-decay scenario*, wherein logging residue decays much faster due to mechanical maceration; 4) *a scatter-and-prescribed burn scenario*, wherein logging residue is inefficiently combusted on site, leaving behind substantial amounts of charcoal and unburned wood; 5) *a scatter-and-wildfire burn scenario*, wherein logging residue is subject to 10% annual probability of experiencing low-severity wildfire; and 6) *a pile-and-burn scenario*, wherein logging residue is efficiently combusted on site, leaving behind only some charcoal and unburned wood. The specific parameters used to define these alternate baselines are given in Table 1.

**Table 1:** Alternate baseline scenarios.

	Fractional fate of logging residue otherwise recoverable as biochar feedstock				
	(proportion of total subject to each condition)				
Baseline scenario	Unmodified and piled	Unmodified and scattered	Chipped scattered	Combusted on site	Converted to charcoal on site
Pile and decay	1.00	0	0	0	0
Scatter and decay	0	1.00	0	0	0
Mulch and decay	0	0.00	1.00	0	0
Pile and prescribe burn	0.05	0	0	0.94	0.01
Scatter and prescribe burn	0	0.74	0	0.25	0.01
Scatter and wild burn*	0	1.0 (initial)	0	0.05 (annual)	0.001 (annual)
Decay constant by pool (yr-1)	0.03	0.05	0.1	na	0.003

* in this scenario, crudely representing a return to very frequent natural fire, all logging residue first enters the unmodified and scattered pool, after which it experiences a 10% annually probability of wildfire, wherein 50% of the debris is combusted and 1% is converted to on-site charcoal.

### Case studies

As part of a separate feasibility analysis [28], 12 different biochar production facilities were conceived for operation in Southern Oregon and Northern California. Each were engineered to process feedstock (i.e. logging residue) from geographically specified sites throughout the Fremont and Winema National Forests, wherein tree thinning is believed to have the largest effect on reducing the flame length and spread rate of future wildfire [29]. These 12 case studies comprise a factorial combination of: facility location (either in Warden, OR or Yreka, CA), pyrolyzer type (conventional thermal or microwave), whether feedstock is dried using solely natural gas or heat recovered from the pyrolyzer, and whether or not heat recovered from the pyrolyzer is used to cogenerate excess electricity. The specific energy and subsequent carbon budgets for each of these production scenarios is detailed in Table 2. In all scenarios, facilities were engineered to process 45.4 Gg of dry feedstock per year and simulated to run for 20 years (crudely approximating the effective life-span of forest fuel reduction treatments and the capital return of plant buildout).

**Table 2:** Estimated hourly energy requirements to process 6.87 Mg of dry feedstock collected from logging sites located in the Fremont-Winema National Forest for various biochar plant configurations and locations.

Biochar plant configuration and location		Diesel fuel to deliver feedstock (liters)	Diesel fuel to handle feedstock (liters)	Diesel fuel to deliver biochar (liters)	Natural gas to dry feedstock (GJ)	Natural gas to run plant (GJ)	Electricity to run plant (GJ)	Electricity produced in co-gen (GJ)
*Thermal pyrolyzer*, *without energy or by product recovery systems*								
	Worden	36	79	9	4.3	26.5	3.2	0
	Yreka	73	79	18	4.3	26.5	3.2	0
*Thermal pyrolyzer*, *paired with heat recovery systems*								
	Worden	36	79	9	0.0	0.5	3.8	0
	Yreka	73	79	18	0.0	0.5	3.4	0
*Thermal pyrolyzer*, *paired with heat and power recovery systems*								
	Worden	36	79	9	0.0	0.5	4.3	5.4
	Yreka	73	79	18	0.0	0.5	4.0	5.4
*Microwave pyrolyzer*, *without energy or by product recovery systems*								
	Worden	36	79	9	8.2	0.0	14.5	0
	Yreka	73	79	18	8.2	0.3	14.5	0
*Microwave pyrolyzer*, *paired with heat recovery systems*								
	Worden	36	79	9	0.0	0.3	15.1	0
	Yreka	73	79	18	0.0	0.3	14.7	0
*Microwave pyrolyzer*, *paired with heat and power recovery systems*								
	Worden	36	79	9	0.0	0.3	15.6	5.4
	Yreka	73	79	18	0.0	0.3	15.3	5.4

6.87 Mg of dry feedstock processed per hour x 6,600 operational hours per year = 45.3 Gg of feedstock processed per year, which at a carbon concentration of 0.5 translates to 22.7 Gg of carbon. Diesel fuel is assumed to release 372 g C per liter consumed, natural gas is assumed to release 14.47 kg C per MMbtu consumed, and locally available electricity is assumed to release 13.7 g C per MJ consumed (US Energy Information Administration). Differences in delivery costs between the Worden and Yreka sites reflect distance to logging sites (providing feedstock), and croplands (where biochar is applied).

## Results and discussion

### Describing basic system behavior and sensitivity to biophysical parameters

The basic dynamics that afford carbon storage in biochar are most easily illustrated by comparing a single pool of decomposing biochar relative to a single pool of unmodified feedstock (Fig 1A and 1B). So long as the production of biochar involves the combustion of some feedstock carbon, doing so results in an initial carbon debt relative to a baseline wherein the feedstock was left unmodified to decay. However, provided that the biochar decomposes slower than the unmodified feedstock, there is inevitably a time at which the pool of slowly decomposing biochar carbon is equal in mass to what the initial feedstock carbon would have been if left unmodified to decay for the same period; we refer to this time point as the compensation point. Beyond the compensation point, the net carbon storage attributed to biocharring increases, until eventually the amortized carbon storage equals the amortized debt incurred prior to the compensation point; we refer to this time point as climate parity. Only after climate parity can it be said that net climate change mitigation is occurring.

While simulating decay from single pools of biochar and residue effectively illustrates the underlying dynamics, a more appropriate evaluation of biochar production comes from a model system wherein residue is continually added and converted to biochar (Fig 1C and 1D).

In this continuous-input system, representing the behavior of an operating biochar facility processing a steady input of common feedstock, dynamic pools of logging residue or biochar accumulate toward their own dynamic equilibrium (Fig 1C). Due to the repeated incursion of carbon debt by continuous biocharring, the compensation point and climate parity are postponed well beyond that achieved by a single input pool. However, after the compensation point is achieved, the net carbon storage attributed to continued biocharring rises almost linearly for several hundred years (Fig 1C and 1D).

**Fig 2:**
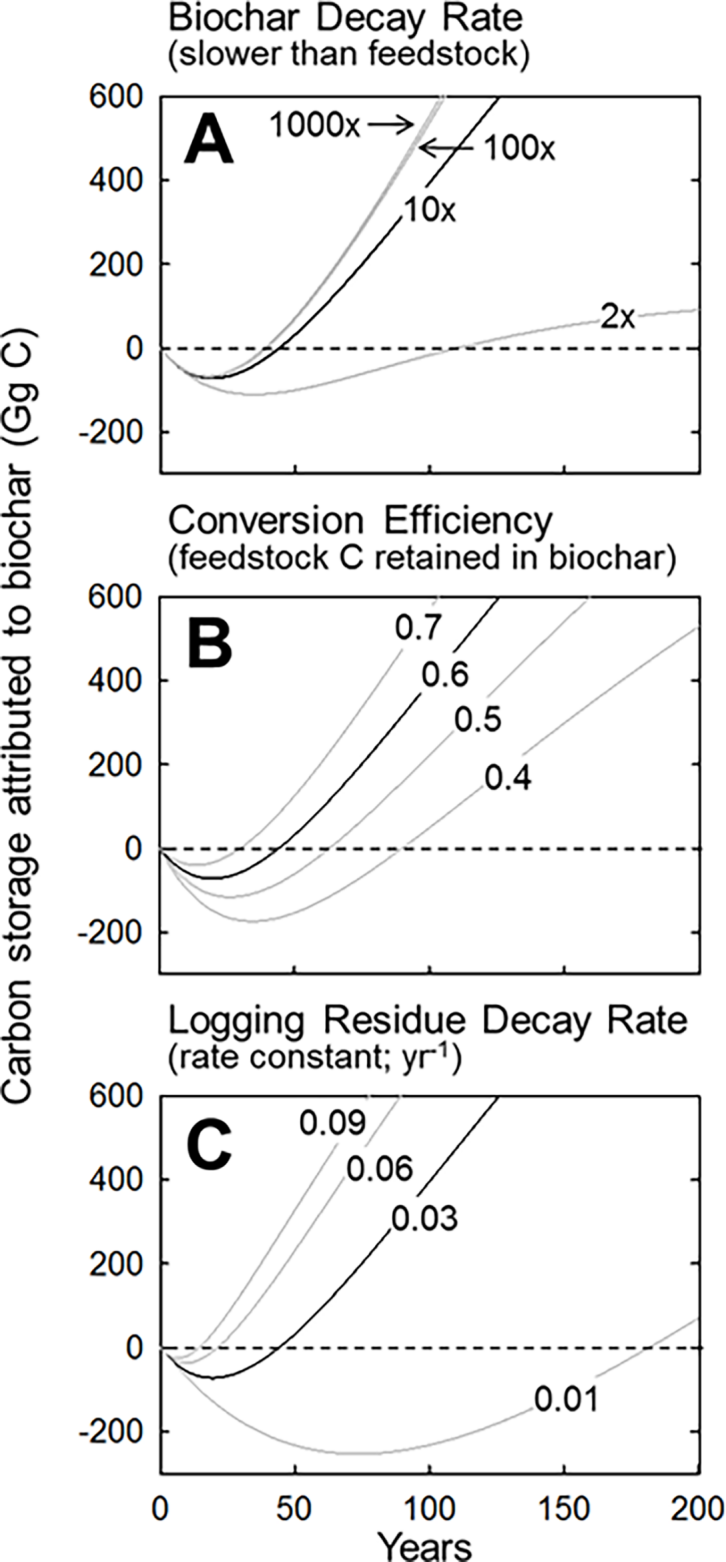
Sensitivity of carbon storage to key biochar biophysical parameters. Relative decay rate (A), carbon conversion efficiency (B), and logging residue decay rate (C). All parameters other than those being varied in each panel were held at common default values, identified in the other panels (here and in Fig 3) as the black line. Most notable is the insensitivity of carbon storage to biochar decay rate, provided it is at least 10-times more recalcitrant than unmodified logging residue.

It is easy to envision the differential decay rates between biochar and unmodified feedstock being critically important in defining the time required for a biocharring operation to reach its carbon compensation point and climate parity. However, these metrics of net carbon storage are remarkably insensitive to biochar recalcitrance provided it is at least 10 times greater than its feedstock. For example, increasing the recalcitrance of biochar from 10 to 1000 times greater than logging residue reduced compensation points only from 44 to 39 years, and climate parity time only from 70 to 61 years (Fig 2A, S1 Table). Biochar recalcitrance does, over many centuries, dictate the size at which the biochar pool equilibrates, but provided it is at least 10 times greater than its feedstock, the rate at which carbon accumulates (in biochar relative to feedstock) over the first 500 years of production is remarkably constant. These results are especially convenient from a carbon accounting perspective since accurately predicting the recalcitrance of various biochars has proven difficult, with crude empirical estimates of residence time being only weakly correlated with composition and realized longevity more likely being controlled by the decay environment [30]. The containment of biochar in dedicated landfills would certainly afford the most reliable verification of its longevity; however, large-scale production of biochar currently depends on demand by agricultural end-users whose applications vary widely from simple soil surface application, to deep tilling, to incorporation of biochar with artificial fertilizers and organic composts. Uncertainty in the fate of biochar after its sale has been a barrier in verifying the carbon storage benefits. Our results suggest that uncertainty in these fates may not profoundly influence carbon storage potential in the first few centuries, so long as most of the biochar remains at least 10-times more recalcitrant than the feedstock from which it was made.

Unlike the relative recalcitrance of biochar, its conversion efficiency (i.e. the fraction of initial feedstock carbon retained in biochar after pyrolysis) strongly affects the time required to reach the compensation point and climate parity (Fig 2B, S1 Table). Using our default decay constants of 0.02 and 0.0002 yr^-1^ for residue and biochar, respectively, an increase in conversion efficiency from 0.4 to 0.7 reduced the climate parity time from 201 years to 46 years. This result also is especially convenient from a carbon accounting perspective since, unlike long-term decay rates, the conversion efficiency of biochar is both easily quantifiable and easily manipulated in a biochar production facility.

Naturally, the longer it would have taken unmodified logging residue to decay on site, the longer it will take for an aggrading pool of biochar, made from that logging residue, to realize its relative carbon storage benefits. Assuming a conversion efficiency of 0.6 and a differential decay rate (between biochar and logging residue) of 10 times, a decrease in logging residue decay rate from 0.01 to 0.09 yr^-1^ can increase climate parity time nearly three centuries from 22 years to 305 years (Fig 2C, S1 Table). This sensitivity of climate parity time to the longevity of logging residue is profound, especially considering how difficult it is to accurately predict in situ decay rates. Factors affecting the decay rate of woody debris include fragment diameter, wood species, microclimate, and soil contact [31] and it is reasonable to assume that decay rates of woody debris in the semi-arid conifer forests most often targeted for fuels reduction are lower than they are in forests experiencing warmer winters and or cooler summers. However, considering how widely empirical estimates of decay rates vary, even within similar forest types, it remains difficult to incorporate these known controls on wood decay into predictive models. For example, observed decay rates for branches and small-diameter boles (of which logging residue is largely comprised) in mixed conifer forests of southern and eastern Oregon (akin to the case studies considered in this study) range between 0.01 to 0.1 yr^-1^ [24,25], respectively. More precise estimates of carbon storage over time, could also be afforded through the use of second-order or even third-order decay functions designed to reflect lags in wood decay (occurring prior to decomposer colonization) and or decelerating decay (as the proportion labile fractions are reduced). However, the information necessary to parameterize such models is lacking and, when tested, first-order decay constants are reasonably accurate in describing mass loss over the first several decades of decay [32, 33]. The use of first-order decay rates between 0.01 to 0.09 yr-1, served the purpose of our sensitivity analysis, but unless one can accurately predict the decay rate of logging residue for any given biochar project, the exact timing of carbon storage benefits will remain uncertain.

### Sensitivity to biochar production parameters

**Fig 3:**
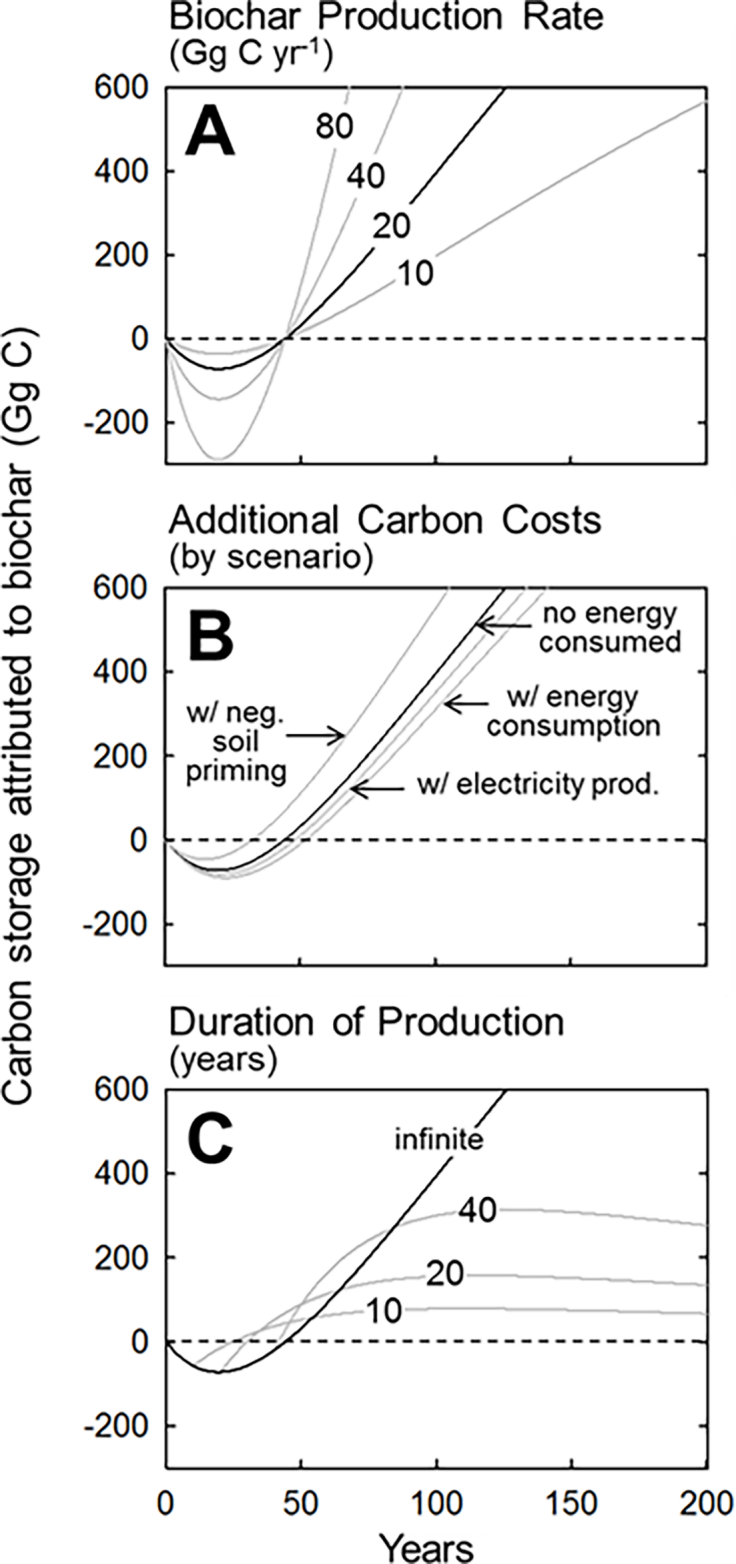
Sensitivity of carbon storage to key biochar production parameters. Production rate (A), additional carbon costs and benefits of production (B), and duration of production (C). All parameters other than those being varied in each panel were held at common default values, identified in the other panels (here and in Fig 2) as the black line. Note the irrelevance of production rate to either compensation point or climate parity, and the relative insensitivity of carbon storage to energy consumption by the production facility.

For the most part, the energy required to produce biochar is derived from the feedstock itself. As such, the bulk of carbon emitted by a biochar facility is accounted for when the pool of feedstock carbon is reduced to a smaller pool of biochar carbon. Nevertheless, a full carbon accounting of biochar facilities should include any additional carbon costs or gains of operation. An evaluation of several hypothetical facilities designed to produce biochar from logging residue (Table 2) indicates that the diesel, natural gas, and electricity consumed in feedstock transportation and preparation can result in additional carbon emissions amounting to 4% of the carbon entering the facility as feedstock. While the cost of purchasing this energy is obviously important to a facility’s operation budget, its consumption has surprisingly little influence on the long-term carbon consequences of biocharring- only scantly increasing the compensation points and climate parity times beyond that when such costs are omitted (Fig 3B, S1 Table). Moreover, facilities equipped with co-generation capacity (converting excess heat from pyrolysis into electricity) can potentially reduce the net carbon costs of operation from 4% to 2% (Fig 3B, S1 Table). These results indicate that the carbon consequences of converting logging residue into biochar (i.e. the initial carbon debt and eventual carbon gain over decades) is shaped almost entirely by the decay rates of feedstock and biochar and the carbon lost in converting the former to the latter. The additional carbon costs of facility operation, and potential gains through energy production, are small by comparison.

The effects of biochar production rate and duration on long-term carbon storage are as one might expect. Specifically, the production rate (i.e. the amount of logging residue processed into biochar each year) has no effect on either the time required to reach the compensation point or climate parity (Fig 3A). This is because smaller biochar operations incur less initial carbon debt but pay that debt back proportionally slower than do larger ones. By comparison, the consequence of production duration on carbon storage is somewhat enigmatic (Fig 2C). Because the running incursion of carbon debt ceases the moment a biochar facility closes, short-duration operations realize their compensation points and climate parities earlier than do longer-duration operations. However, by producing more biochar, longer-duration operations store greater amounts of carbon over time. Notably, when using our best estimates of conversion efficiency and decay rates, producing biochar from logging residue continuously for 40 years stores carbon for the first 100 years in a manner very similar to a perpetually running operation (Fig 3C, S1 Table).

### Alternate baselines

**Fig 4:**
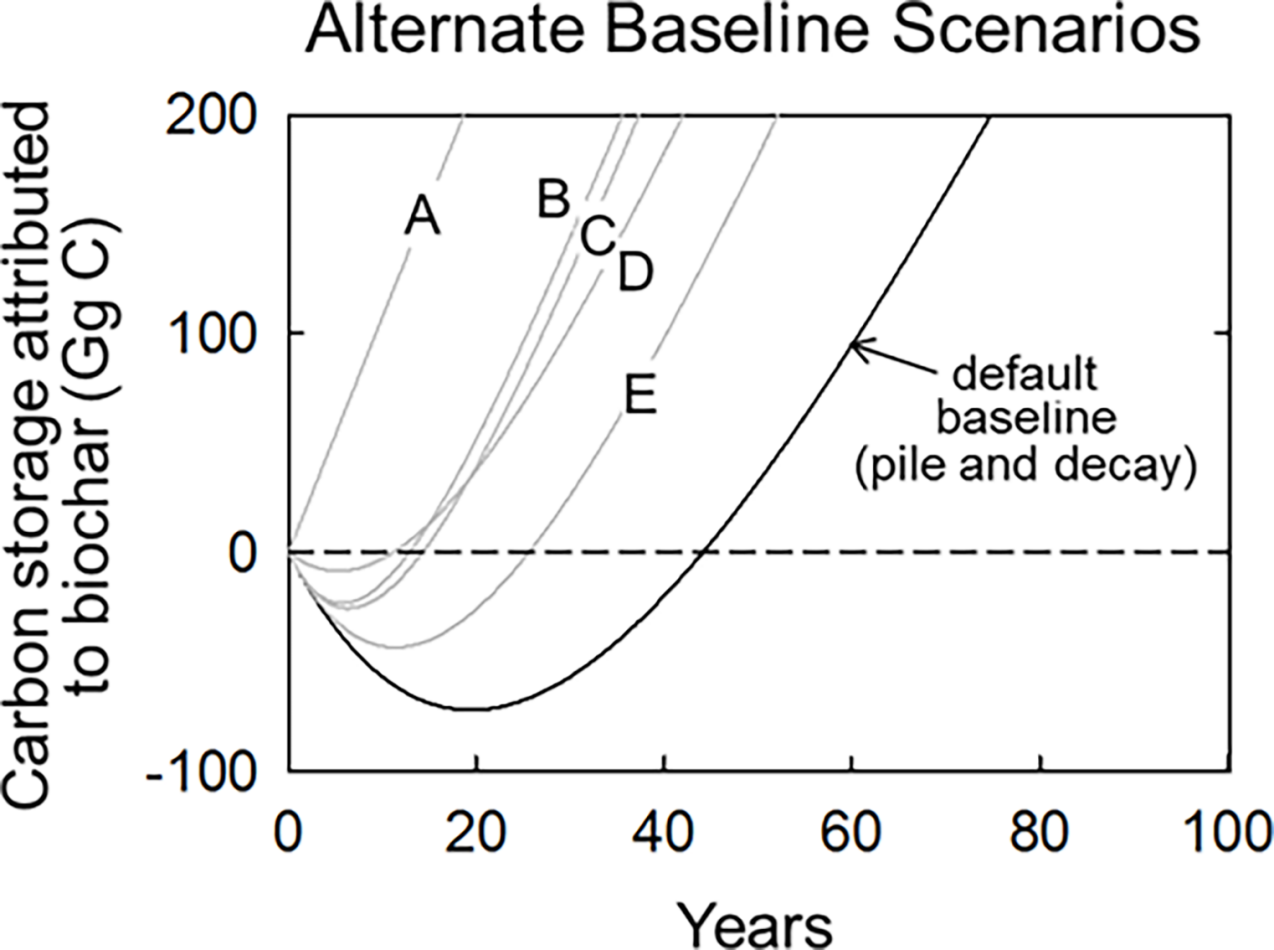
Carbon storage in biochar relative to different baseline scenarios, each defined by an alternate fate of logging residue otherwise recoverable as biochar feedstock. *Pile and decay* (default), where residue is piled and allowed to decay on site; *pile and burn* (A), where residue is piled and burned on site to near completion within one year; *scatter and wildfire* (B), where residue is scattered on site and then subject to wildfire at a 10 year probabilistic return interval; *mulch and decay* (C), where residue is chipped, scattered, and allowed to decay on site; *scatter and burn* (D), where residue is scattered on site and subject to controlled burning within one year; and *scatter and decay* (E), where residue is scattered and allowed to decay on site.

Among the various on-site fates of logging residue, the pile-and-decay scenario results in the slowest carbon emission rates. As such, the relative storage of carbon attributable to biocharring increases when baselines involve greater contact between logging residue and the soil and/or combustion (Fig 4). For instance, when biocharring is compared to a *scatter-and-decay* baseline, wherein decomposition is accelerated without combustion, the carbon compensation point drops from 45 years to about 25 years (reflecting the same system sensitivity to residue decay rate shown in Fig 2B). The relative storage of carbon attributable to biocharring increases even more when compared to *mulch-and-decay*, *scatter-and prescribe burn*, *and scatter-and-wild burn* scenarios, all of which result in carbon compensation points of around 15 years and climate parity times of around 25 years. When biocharring is compared to a *pile-and-burn* baseline, wherein logging residue is nearly completely combusted on site, net storage begins immediately and the compensation point becomes zero. When using this *pile-and-burn* baseline, biocharring incurs no carbon debt, and patterns of carbon storage over time are similar to that achieved through biocharring short-lived crop residues.

Which of these baselines is most appropriate when crediting biochar for carbon storage? So long as the logging prescription specifically calls for the burning of residue as a necessary means to reduce fire hazard, a reasonable case could be made for the use of a *pile-and-burn* baseline, or at least a *scatter-and-burn* baseline. Indeed, there is a large body of literature suggesting that critical reductions in wildfire intensity (i.e. flame height and spread rates most associated with overstory mortality and undesirable soil impacts) are most often achieved when thinning operations are followed up with prescribed burning of residue [34]. However, operational costs of prescribed burning, regulations on smoke pollution, and concerns over fire escape often result in some combination of the *pile-and-decay* and *lop-and-scatter* approach for residue management. Fully accounting for the fate of logging residue theoretically should also include some probabilistic exposure to wildfire (e.g. the historic 10% annual fire frequency simulated in our *scatter-and-wild burn* scenario). However, as of yet, fire suppression remains the prevailing management approach to wildfire for most forests in North America, rendering the likelihood of any logging residue experiencing wildfire of any severity typically less than 0.5% annually [35,36] resulting in residue residence times not that different from no burning at all.

One may also consider an accounting scheme that extends to include the influence of thinning on the carbon stored in the forest landscape. Under such a scheme, the carbon storage attributed to biocharring could partially offset the losses in forest biomass necessarily associated with maintaining a fire-resilient landscape through thinning [37]. Regardless of how carbon markets or regulators might ultimately account for the effects of biocharring logging residue, the most informative evaluation of biochar’s impact on carbon storage comes by comparing it to a simple and commonly employed no-action, leave-and-decay baseline.

### Case studies

**Fig 5:**
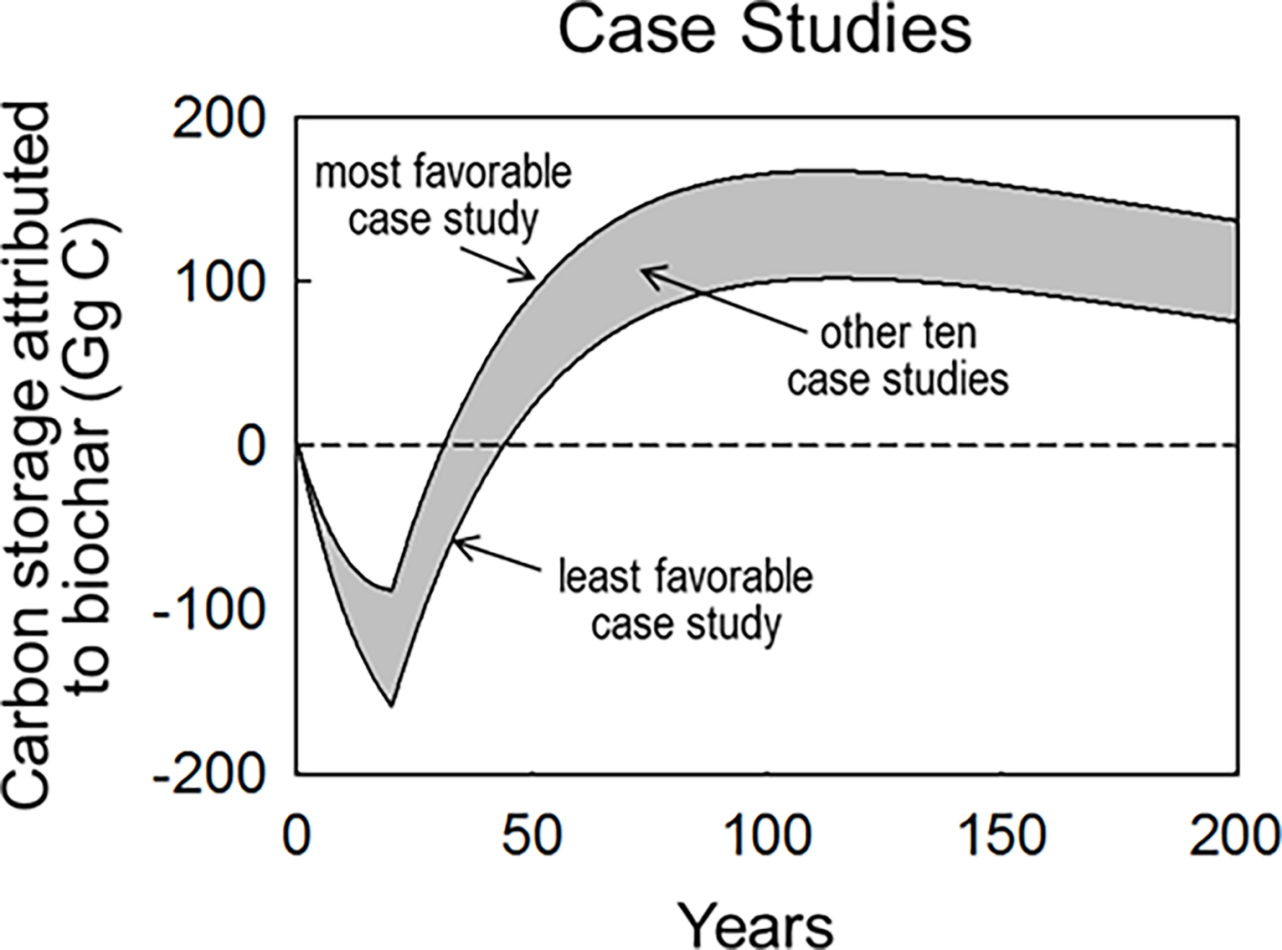
Carbon storage achieved by 12 theoretical biochar production facilities comprising a factorial combination of: Facility location (relative to feedstock source), heat recovery (or not), heat and electricity recovery (or not), and pyrolysis type (thermal or microwave). In each case, facilities processed 23 Gg C yr^-1^ (in the form of logging residue) into biochar for a fixed period of 20 years. Carbon Storage over time is assessed relative to a leave-and-decay baseline. The most favorable case involved microwave pyrolysis, heat and electricity recovery, in the facility nearest the feedstock source. The least favorable case involved thermal pyrolysis, without heat or electricity recovery, in the facility farthest from the feedstock source.

Among the 12 different production facilities conceived to make biochar from logging residue in Southern Oregon for a fixed duration of 20 years, all reached their carbon compensation point (relative to a pile-and-decay baseline) in less the 45 years, and achieved climate parity in less the 100 years (Fig 5, S2 Table). Moreover, the carbon storage attributed to biocharring in these facilities continued to increase for about a century (Fig 5). For most plant configurations, the conversion of nearly 500 Gg of logging residue carbon into biochar, over a period of 20 years, resulted in a 200 year mean storage of approximately 100 Gg of C above what would have been stored in that same logging residue if left to decay on site (S2 Table). As expected, facilities located farther from the logging sites and designed without either heat or electrical co-generation capacity resulted in less carbon storage over time than did facilities located closer to feedstock sources and requiring less external energy. More notable than these anticipated differences was just how conserved net carbon storage was among facility configurations (Fig 5). As the sensitivity analysis shown earlier in this study reveals, carbon storage in biochar relative to unmodified logging residue is dictated much more by conversion efficiency during pyrolysis (Fig 2B), which was conserved among our case studies, than it is by facility-specific energy consumption (Fig 3B).

## Conclusions

### Carbon market compatibility

As demonstrated here, the deliberate conversion of logging residue into highly recalcitrant biochar serves eventually to increase terrestrial carbon storage beyond what would occur if this woody residue was left in the forest to decay and or combust. However, recognition of this storage potential by existing carbon regulatory schemes and or carbon markets has proven difficult. The only biochar activities currently recognized by regulators as carbon positive are those that result in immediate and quantifiable emission reduction, such as increasing conversion efficiencies within existing charcoal production facilities (i.e. more charcoal produced per feedstock combusted) [38]. As of yet, efforts to assign carbon credits to biochar made from agricultural residues, including logging residues, have been rejected due largely to uncertainties in the decay rates of biochar [39]. It is necessarily the case that even small variations in biochar decomposition rates will influence the size at which any dynamic pool of biochar will eventually equilibrate. However, according to our analysis, the rate at which this equilibrium is initially approached, and hence the net storage realized in the first two centuries of production, is largely insensitive to biochar decomposition rates. So long as carbon crediting schemes do not exceed a 100-year time horizon, and biochar is at least ten-times more recalcitrant than the feed stock they are made from, further uncertainty in its realized decay rate may not be the barrier to verification it is currently believed to be.

A proper carbon crediting scheme for biochar production from logging residue would also need to address the substantial time lag between project initiation and realized storage. It is easy to imagine an ex-ante crediting scheme that could discount long-term carbon gains by the short-term carbon losses of biochar production, but any such accounting should also acknowledge that a mass of carbon emitted today is not equivalent to that same mass of carbon sequestered a century from now. After all, planetary responses to rising global temperatures may not be quickly reversed by future reductions in greenhouse gas concentrations.

### Permanence, leakage, and additionality

Despite accounting complications arising from inherent storage lags, the carbon benefits of converting logging residue into biochar easily meet other key crediting criteria, such as permanence, leakage, and additionality. Regarding permanence: while the realized longevity of biochar in various substrates remains uncertain, our analyses indicate sustained storage–above baseline scenarios for centuries–across a wide range of biochar half-lives. In this respect, carbon storage in biochar derived from logging residue exhibits high permanence provided it finds itself in an environment wherein it will decay at least ten-times slower than would the feedstock it was made from. Regarding leakage: unlike actions that curtail the production of commodities to save carbon, the conversion of logging residue into biochar does not create a market incentive to release carbon elsewhere; moreover, logging activities conducted to restore desirable forest structure function only to reduce, not increase, demand for wood from other forests, rendering leakage negative. In this respect, carbon storage in biochar derived from logging residue exhibits no positive leakage. Regarding additionality: Except in cases where forest thinning actually averts a permanent, climate-driven transition to non-forest [40], the structural changes induced by forest thinning necessarily reduce long-term carbon storage in the forest and in wood products [37]. The analysis presented in this study starts with the assumption that a forest has been thinned and evaluates only the carbon storage advantage of making biochar out of the unmerchantable logging residue versus not doing so. As such, the full carbon costs of maintaining low-density, fire resilient forests have not been included in our analysis. However, we contend that since the ecological, social, and economic forces motivating the thinning of fire-prone forests exist independent of biochar production, it is reasonable to consider residue from these logging prescriptions as a *bona fide* byproduct. In this respect, carbon storage in biochar should meet standards of additionality, even when the activities generating the feedstock are themselves carbon negative.

## Supporting information

S1 Table Carbon storage in biochar made from logging residue relative to a baseline where unmodified logging residue decays on site. Residue decay rate is the natural-log, first-order decay constant of unmodified logging residue. Differential decay is the factor by which biochar made from logging residue, decays slower than unmodified logging residue. Conversion efficiency is the fraction of logging residue carbon retained in biochar made by pyrolysis of that logging residue. Consumption rate is the mass of logging residue carbon (feedstock) consumed per year to make biochar. Production duration is the number of years logging residue is converted into biochar. C costs of production is the net carbon released to the atmosphere in: feedstock transportation, feedstock handling, feedstock drying, biochar end-use transportation and soil incorporation, fossil fuel offsets attributed to electricity returned to power grid, and soil priming effects of biochar on native soil organic matter per feedstock carbon processed into biochar. Compensation point is when the carbon stored in biochar is equal to that which would have been stored in logging residue, if left unmodified. Climate parity is when the amortized carbon storage attributed to biochar equals the amortized carbon debt incurred prior to the compensation point. 100 yr, 200 yr, and 400 yr mean storage is the average net carbon storage (in soil-incorporated biochar relative to a baseline where unmodified logging residue decays on site) over a period of 100, 200, and 400 years, respectively.
(PDF)

S2 Table Long-term carbon storage achieved by 12 theoretical biochar production facilities. C costs of production is the net carbon released to the atmosphere in: feedstock transportation, feedstock handling, feedstock drying, biochar end-use transportation and soil incorporation, fossil fuel offsets attributed to electricity returned to power grid, and soil priming effects of biochar on native soil organic matter per feedstock carbon processed into biochar. Compensation point is when the carbon stored in biochar is equal to that which would have been stored in logging residue, if left unmodified. Climate parity is when the amortized carbon storage attributed to biochar equals the amortized carbon debt incurred prior to the compensation point. 100 yr, 200 yr, and 400 yr mean storage is the average net carbon storage (in soil-incorporated biochar relative to a baseline where unmodified logging residue decays on site) over a period of 100, 200, and 400 years, respectively. Residue decay rate (the natural-log, first-order decay constant of unmodified logging residue) = 0.03; differential decay (the factor by which biochar made from logging residue, decays slower than unmodified logging residue) = 10x; conversion efficiency (the fraction of logging residue carbon retained in biochar made by pyrolysis of that logging residue) = 0.6 and 0.65 for thermal and microwave pyrolysis, respectively; consumption rate (the mass of logging residue carbon consumed to make biochar) = 23 Gg C yr-1. Production duration (the period that logging residue is converted into biochar) = 20 yr.
(PDF)
