# Supplementary material for: Potential carbon storage in biochar made from logging residue: Basic principles and Southern Oregon case studies
Source: PLoS One. 2018 Sep 13;13(9):e0203475. doi: 10.1371/journal.pone.0203475 (PMC6136743; doi:10.1371/journal.pone.0203475)
Supplement: S1 Table — Residue decay rate is the natural-log, first-order decay constant of unmodified logging residue. Differential decay is the factor by which biochar made from logging residue, decays slower than unmodified logging residue. Conversion efficiency is the fraction of logging residue carbon retained in biochar made by pyrolysis of that logging residue. Consumption rate is the mass of logging residue carbon (feedstock) consumed per year to make biochar. Production duration is the number of years logging residue is converted into biochar. C costs of production is the net carbon released to the atmosphere in: feedstock transportation, feedstock handling, feedstock drying, biochar end-use transportation and soil incorporation, fossil fuel offsets attributed to electricity returned to power grid, and soil priming effects of biochar on native soil organic matter per feedstock carbon processed into biochar. Compensation point is when the carbon stored in biochar is equal to that which would have been stored in logging residue, if left unmodified. Climate parity is when the amortized carbon storage attributed to biochar equals the amortized carbon debt incurred prior to the compensation point. 100 yr, 200 yr, and 400 yr mean storage is the average net carbon storage (in soil-incorporated biochar relative to a baseline where unmodified logging residue decays on site) over a period of 100, 200, and 400 years, respectively. (PDF) [file pone.0203475.s001.pdf]

Table S1. Carbon storage in biochar made from logging residue relative to a baseline where unmodified logging residue decays on site.

residue decay rate (yr^{-1})	differential decay (factor)	conversion efficiency (fraction)	consumption rate (Gg C yr^{-1})	production duration (years)	C costs of production (fraction)	compensation point (years)	climate parity (years)	100yr mean storage (Gg C)	200yr mean storage (Gg C)	400yr mean storage (Gg C)
Sensitivity to differential decay, under a perpetual production scenario										
0.03	2x	0.6	20	1000	0	110	227	-73	-13	52
0.03	10x	0.6	20	1000	0	44	70	88	436	1058
0.03	100x	0.6	20	1000	0	39	61	139	622	1697
0.03	1000x	0.6	20	1000	0	39	61	144	643	1781
Sensitivity to conversion efficiency, under a perpetual production scenario										
0.03	10x	0.4	20	1000	0	90	201	-97	102	498
0.03	10x	0.5	20	1000	0	63	103	-5	269	778
0.03	10x	0.6	20	1000	0	44	70	88	436	1058
0.03	10x	0.7	20	1000	0	30	46	180	603	1338
Sensitivity to feedstock consumption rate, under a perpetual production scenario										
0.03	10x	0.6	10	1000	0	44	70	44	218	529
0.03	10x	0.6	20	1000	0	44	70	88	436	1058
0.03	10x	0.6	40	1000	0	44	70	176	871	2116
0.03	10x	0.6	80	1000	0	44	70	351	1743	4233
Sensitivity to decay rate of unmodified logging residue, under a perpetual production scenario										
0.09	10x	0.6	20	1000	0	14	22	347	782	1453
0.06	10x	0.6	20	1000	0	21	33	267	686	1350
0.03	10x	0.6	20	1000	0	44	70	88	436	1058
0.01	10x	0.6	20	1000	0	182	305	-196	-146	159
Sensitivity to differential decay, under a fixed 20-year production scenario										
0.03	2x	0.6	20	20	0	45	120	-6	9	6
0.03	10x	0.6	20	20	0	30	53	65	107	104
0.03	100x	0.6	20	20	0	28	48	89	155	187
0.03	1000x	0.6	20	20	0	28	48	92	161	199
Sensitivity to conversion efficiency, under a fixed 20-year production scenario										
0.03	10x	0.4	20	20	0	45	99	2	49	58
0.03	10x	0.5	20	20	0	37	72	33	78	81
0.03	10x	0.6	20	20	0	30	53	65	107	104
0.03	10x	0.7	20	20	0	24	39	97	136	127
Sensitivity to feedstock consumption rate, under a fixed 20-year production scenario										
0.03	10x	0.6	10	20	0	30	53	33	54	52
0.03	10x	0.6	20	20	0	30	53	65	107	104
0.03	10x	0.6	40	20	0	30	53	130	214	209
0.03	10x	0.6	80	20	0	30	53	260	428	417
Sensitivity to decay rate of unmodified logging residue, under a fixed 20-year production scenario										
0.09	10x	0.6	20	20	0	14	22	144	151	126
0.06	10x	0.6	20	20	0	21	30	123	140	121
0.03	10x	0.6	20	20	0	30	53	65	107	104
0.01	10x	0.6	20	20	0	84	191	-48	4	40
Sensitivity to production duration										
0.03	10x	0.6	20	10	0	25	49	36	55	53
0.03	10x	0.6	20	20	0	30	53	65	107	104
0.03	10x	0.6	20	40	0	42	62	100	200	205
0.03	10x	0.6	20	80	0	44	70	99	343	393
Sensitivity to net additional carbon costs of production, under a perpetual production scenario										
0.03	10x	0.6	20	1000	0.00	44	70	88	436	1058
0.03	10x	0.6	20	1000	0.04	52	84	44	350	887
0.03	10x	0.6	20	1000	0.02	48	77	65	390	968
0.03	10x	0.6	20	1000	0.12	71	119	-37	188	566
0.03	10x	0.6	20	1000	-0.08	32	50	167	592	1370
Sensitivity to net additional carbon costs of production, under a fixed 20-year production scenario										
0.03	10x	0.6	20	20	0.00	30	53	65	107	104
0.03	10x	0.6	20	20	0.04	33	61	50	91	88
0.03	10x	0.6	20	20	0.02	31	57	57	98	96
0.03	10x	0.6	20	20	0.12	39	81	21	60	56
0.03	10x	0.6	20	20	-0.08	25	41	93	137	135

Residue decay rate is the natural-log, first-order decay constant of unmodified logging residue. *Differential decay* is the factor by which biochar made from logging residue, decays slower than unmodified logging residue. *Conversion efficiency* is the fraction of logging residue carbon retained in biochar made by pyrolysis of that logging residue. *Consumption rate* is the mass of logging residue carbon (feedstock) consumed per year to make biochar. *Production duration* is the number of years logging residue is converted into biochar. *C costs of production* is the net carbon released to the atmosphere in: feedstock transportation, feedstock handling, feedstock drying, biochar end-use transportation and soil incorporation, fossil fuel offsets attributed to electricity returned to power grid, and soil priming effects of biochar on native soil organic matter per feedstock carbon processed into biochar. *Compensation point* is when the carbon stored in biochar is equal to that which would have been stored in logging residue, if left unmodified. *Climate parity* is when the amortized carbon storage attributed to biochar equals the amortized carbon debt incurred prior to the compensation point. *100 yr, 200 yr, and 400 yr mean storage* is the average net carbon storage (in soil-incorporated biochar relative to a baseline where unmodified logging residue decays on site) over a period of 100, 200, and 400 years, respectively.
