# Supplementary material for: Potential carbon storage in biochar made from logging residue: Basic principles and Southern Oregon case studies
Source: PLoS One. 2018 Sep 13;13(9):e0203475. doi: 10.1371/journal.pone.0203475 (PMC6136743; doi:10.1371/journal.pone.0203475)
Supplement: S2 Table — C costs of production is the net carbon released to the atmosphere in: feedstock transportation, feedstock handling, feedstock drying, biochar end-use transportation and soil incorporation, fossil fuel offsets attributed to electricity returned to power grid, and soil priming effects of biochar on native soil organic matter per feedstock carbon processed into biochar. Compensation point is when the carbon stored in biochar is equal to that which would have been stored in logging residue, if left unmodified. Climate parity is when the amortized carbon storage attributed to biochar equals the amortized carbon debt incurred prior to the compensation point. 100 yr, 200 yr, and 400 yr mean storage is the average net carbon storage (in soil-incorporated biochar relative to a baseline where unmodified logging residue decays on site) over a period of 100, 200, and 400 years, respectively. Residue decay rate (the natural-log, first-order decay constant of unmodified logging residue) = 0.03; differential decay (the factor by which biochar made from logging residue, decays slower than unmodified logging residue) = 10x; conversion efficiency (the fraction of logging residue carbon retained in biochar made by pyrolysis of that logging residue) = 0.6 and 0.65 for thermal and microwave pyrolysis, respectively; consumption rate (the mass of logging residue carbon consumed to make biochar) = 23 Gg C yr-1. Production duration (the period that logging residue is converted into biochar) = 20 yr. (PDF) [file pone.0203475.s002.pdf]

Table S2. Long-term carbon storage achieved by 12 theoretical biochar production facilities.

biochar plant configuration and location	C costs of production (fraction)	compensation point (years)	climate parity (years)	100yr mean storage (Gg C)	200yr mean storage (Gg C)	400yr mean storage (Gg C)
Thermal pyrolyzer, without energy or by product recovery systems						
Worden	0.16	43	93	8	52	47
Yreka	0.17	44	97	4	48	43
Thermal pyrolyzer, paired with heat recovery systems						
Worden	0.04	33	61	57	103	100
Yreka	0.05	33	63	53	100	96
Thermal pyrolyzer, paired with heat and power recovery systems						
Worden	0.03	32	58	63	111	107
Yreka	0.03	32	59	60	107	103
Microwave pyrolyzer, without energy or by product recovery systems						
Worden	0.11	34	66	47	91	83
Yreka	0.12	35	68	43	86	78
Microwave pyrolyzer, paired with heat recovery systems						
Worden	0.08	32	59	59	104	96
Yreka	0.09	33	61	56	100	92
Microwave pyrolyzer, paired with heat and power recovery systems						
Worden	0.06	31	56	66	111	104
Yreka	0.07	32	58	62	107	100

C costs of production is the net carbon released to the atmosphere in: feedstock transportation, feedstock handling, feedstock drying, biochar end-use transportation and soil incorporation, fossil fuel offsets attributed to electricity returned to power grid, and soil priming effects of biochar on native soil organic matter per feedstock carbon processed into biochar. Compensation point is when the carbon stored in biochar is equal to that which would have been stored in logging residue, if left unmodified. Climate parity is when the amortized carbon storage attributed to biochar equals the amortized carbon debt incurred prior to the compensation point. 100 yr, 200 yr, and 400 yr mean storage is the average net carbon storage (in soil-incorporated biochar relative to a baseline where unmodified logging residue decays on site) over a period of 100, 200, and 400 years, respectively. Residue decay rate (the natural-log, first-order decay constant of unmodified logging residue) = 0.03; differential decay (the factor by which biochar made from logging residue, decays slower than unmodified logging residue) = 10x; conversion efficiency (the fraction of logging residue carbon retained in biochar made by pyrolysis of that logging residue) = 0.6 and 0.65 for thermal and microwave pyrolysis, respectively; consumption rate (the mass of logging residue carbon consumed to make biochar) = 23 Gg C yr-1. Production duration (the period that logging residue is converted into biochar) = 20 yr.
